# The Contribution of Plasma and Brain Vitamin C on Age and Gender-Related Cognitive Differences: A Mini-Review of the Literature

**DOI:** 10.3389/fnint.2020.00047

**Published:** 2020-08-21

**Authors:** Nikolaj Travica, Karin Ried, Irene Hudson, Avni Sali, Andrew Scholey, Andrew Pipingas

**Affiliations:** ^1^Centre for Human Psychopharmacology, Swinburne University of Technology, Melbourne, VIC, Australia; ^2^The National Institute of Integrative Medicine, Melbourne, VIC, Australia; ^3^Discipline of General Practice, University of Adelaide, Adelaide, SA, Australia; ^4^Torrens University, Melbourne, VIC, Australia; ^5^School of Science, College of Science, Engineering and Health, Mathematical Sciences, Royal Melbourne Institute of Technology (RMIT), Melbourne, VIC, Australia; ^6^School of Mathematical and Physical Science, University of Newcastle, Callaghan, NSW, Australia

**Keywords:** ascorbic acid, cognition, central nervous system, dimorphism, aging

## Abstract

There is increasing evidence that sex differences in the brain may contribute to gender-related behavioral differences, including cognitive function. Literature has revealed gender dimorphisms in cognitive function between males and females. Additionally, several risk factors associated with cognitive decline depend on chronological age. It is well recognized that the process of aging is associated with a decline in cognitive ability and brain function. Various explanations may account for these gender-related cognitive differences and age-associated cognitive changes. Recent investigations have highlighted the importance of vitamin C in maintaining brain health and its association with cognitive function in both cognitively intact and impaired cohorts. The present review explores previous literature that has evaluated differences in plasma/brain vitamin C between genders and during aging. It then assesses whether these age and gender-related differences may affect the relationship between plasma/brain vitamin C and cognition. The purpose of this review was to examine the evidence for a link between plasma/brain vitamin C and cognition and the impact of gender and age on this relationship. Epidemiological studies have frequently shown higher vitamin C plasma concentrations in women. Similarly, aging has been systematically associated with reductions in plasma vitamin C levels. A range of animal studies has demonstrated potential gender and age-related differences in vitamin C brain distribution and utilization. The reviewed literature suggests that gender differences in plasma and brain vitamin C may potentially contribute to differences in gender-associated cognitive ability, particularly while females are pre-menopausal. Additionally, we can propose that age-associated differences in plasma and brain vitamin C may be potentially linked to age-associated cognitive differences, with older cohorts appearing more vulnerable to experience declines in plasma vitamin C concentrations alongside compromised vitamin C brain regulation. This review encourages future investigations to take into account both gender and age when assessing the link between plasma vitamin C concentrations and cognitive function. Further large scale investigations are required to assess whether differences in cognitive function between genders and age groups may be causally attributed to plasma vitamin C status and brain distribution and utilization.

## Introduction

For centuries, scientists have been fascinated by differences in gender-related behaviors, particularly cognitive function. Differences in cognitive performance between cognitively intact gender groups have been demonstrated (Linn and Petersen, 1985) with comprehensive meta-analyses revealing that (Hyde, 2005; Lindberg et al., 2010) women outperform men on tasks relating to verbal memory, verbal recognition, and semantic fluency (Linn and Petersen, 1985). While women have higher attention (Liu et al., 2013) and inhibition (Yuan et al., 2008), males tend to demonstrate a higher visuospatial ability (de Frias et al., 2006) and complex visual-spatial episodic memory (Andreano and Cahill, 2009). Numerous explanations have been postulated for the cognitive differences (Gur et al., 1999; Janowsky, 2006; Cosgrove et al., 2007; Kander et al., 2017) but have not been confirmed.

There is a clear age-associated decline in cognitive ability (Murman, 2015). Multiple underlying pathophysiologies that are associated with cognitive decline depend on chronological age (years since birth), many of which arise during midlife (Legdeur et al., 2018). From early adulthood, there are declines in several cognitive domains such as processing speed, reasoning, memory, and executive functions, some of which are underpinned by a decline in a general cognitive function (Deary et al., 2009). Structural and functional changes in the brain correlate with these age-related cognitive changes, including alterations in neuronal structure, loss of synapses, and dysfunction of neuronal networks (Murman, 2015). Although various structural differences have been identified during aging, numerous factors involved in preventing these changes and potentially preserving cognitive decline across the lifespan have become of interest.

Variations in nutritional status, within both blood and brain tissue, amongst different ages and genders may be linked to cognitive changes throughout the life span as well as potentially influence cognitive differences between males and females. This may encompass a variety of nutrients, including vitamin C, which has been studied in both animal models and humans, displaying a possible association with the various vital central nervous system and cognitive functions (Kocot et al., 2017).

The present review highlights the results from previous investigations that have assessed the pharmacokinetics and biological roles of vitamin C on the central nervous system and the link between plasma vitamin C and cognitive function. This is followed by an assessment of studies that have investigated the differences in plasma vitamin C concentrations between genders and during aging. This is followed by an evaluation of whether age and gender may play a role in the relationship between plasma/brain vitamin C and cognition. The purpose of this review was to examine the evidence for an association between plasma/brain vitamin C and cognition which may be affected by gender and age.

### Vitamin C and Its Central Nervous System Functions

A vast array of nutrients have been implemented to play roles in cognitive function (Kidd, 1999; Gómez-Pinilla, 2008), including vitamin C (Travica et al., 2017). Ascorbic acid, the reduced form of vitamin C, is a potent water-soluble antioxidant, not being synthesized in any human tissues, including the brain. Studies have demonstrated the vitamin’s key role in neuromodulation, by being involved as a cofactor for the synthesis of catecholamines (Gupta et al., 2014), serotonin (Gupta et al., 2014) and synaptic release of acetylcholine (Kuo et al., 1979). The vitamin has further been implicated in neurodevelopment (Hansen et al., 2014), stimulation of brain-derived neurotrophic factor (Grant et al., 2005), cardiovascular support through collagen production, angiogenesis, reduced nitric oxide metabolism, and neuronal energy production and sustainability through the biosynthesis of carnitine (Johnston et al., 2006).

Vitamin C’s neuroprotective roles include prevention of neuronal overstimulation by glutamate (excitotoxicity; Qiu et al., 2007); regeneration of other antioxidants such as urate, glutathione, β-carotene, and α-tocopherol (vitamin E; Lykkesfeldt et al., 2007); reduction of lipid peroxidation (May and Qu, 2010); and the reduction of pro-inflammatory cytokines by binding onto nuclear factor kappa B (NF-kB; Bowie and O’Neill, 2000). Both *in vitro* and *in vivo* experiments have supported the vitamin’s crucial role in the brain as a scavenger of reactive oxygen species (ROS; Duarte and Lunec, 2005).

### Vitamin C Transport and Distribution into the Central Nervous System

**Figure 1:**
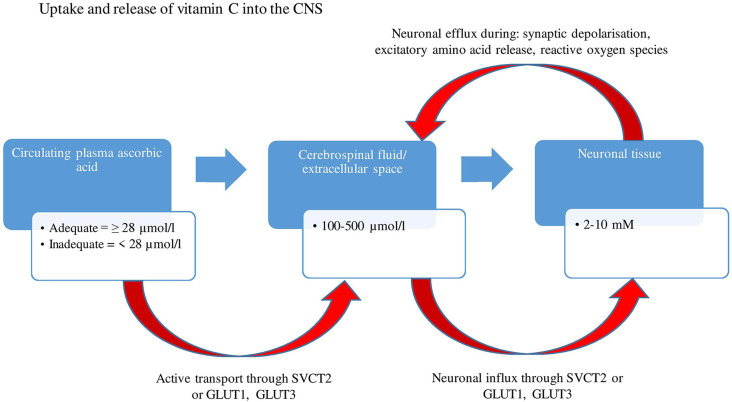
A two-stage uptake mechanism from blood to cerebrospinal fluid (CSF) and from CSF to neuron. Ascorbic acid is transported by an active mechanism from plasma into CSF and from CSF into the neuron through SVCT2, whereas dehydroascorbic acid is transported through GLUT1 and GLUT 3 (glucose receptors). Concentrations of ascorbate range between 100–500 μmol/L within CSF and extracellular space, eventually reaching concentrations as high as 10 mM within neurons. There is an efflux of neuronal ascorbic acid following synaptic activity and secretion of excitatory amino acids. GLUT1, Glucose transporter 1; GLUT3, Glucose transporter 3; SVCT2, Sodium dependent vitamin transporter 2; μmol/L, Micromole per liter; mM, Millimole.

Human autopsy studies have indicated that vitamin C is most abundant in the cerebral cortex, hippocampus, and amygdala (Oke et al., 1987). Ascorbic acid is maintained at concentrations as high as 10 mM in neurons, 100 times higher than those present in the circulating plasma (Rice, 2000), suggestive of the vitamin’s importance in brain functioning. These high concentrations are a result of extracellular dehydroascorbic acid (DHAA-oxidized ascorbate) being recycled by glutathione dependant reductases (e.g., glutathione-disulfide reductase) and nicotinamide adenine dinucleotide phosphate (NADPH), within astrocytes, back into ascorbate and being compartmentalized into neurons and reused intracellularly (Covarrubias-Pinto et al., 2015). Based on animal research, concentrations can change depending on brain activity and circadian rhythms, with studies demonstrating a neuronal efflux and increase in extracellular ascorbate levels following synaptic activity (depolarization), the secretion of excitatory amino acids (glutamate and aspartate; Dodd and Bradford, 1974) and possible co-release with catecholamines (Figure 1).

A range of factors influences CNS vitamin C distribution, including aging that is associated with depletion of vitamin C in brain tissue, especially within the cerebral cortex, pituitary gland, and hippocampus (Schaus, 1957; Siqueira et al., 2011). Additionally, the glucose transporter (GLUT) which also transports DHAA is competitively inhibited by glucose, with excess glucose in plasma or intestine blocking the receptor-binding site and decreasing GLUT-facilitated DHAA transport (Wilson, 2003).

Several studies have examined the relationship between vitamin C concentrations in plasma and the CNS. An active transport mechanism conveys plasma vitamin C predominantly through the choroid plexus, into the cerebrospinal fluid (CSF), where ascorbate concentrations are 2.5–4 times higher than in plasma. From the CSF, ascorbate diffuses into extracellular fluid (ECF) which maintains equal concentrations of ascorbate and provides the ascorbate for neuronal uptake (Rice, 2000). Despite wide fluctuations of plasma ascorbate levels, CSF concentrations have been shown to remain relatively stable (Paraskevas et al., 1997). Under conditions of ascorbic acid deficiency, the brain content of ascorbic acid is retained tenaciously, with decreases of less than 2% per day (Rice, 2000).

A recent *in vivo* study displayed that the choroid plexus is a vital peripheral and brain circadian clock, suggestive of a circadian rhythm of vitamin C availability and homeostasis in the brain (Myung et al., 2018) and that peripheral feedback to the brain may be modulated by circadian time. Additional factors, discussed below may contribute to the distribution of vitamin C within the brain.

Although higher plasma vitamin C concentrations are generally associated with higher CSF levels (Tallaksen et al., 1992), CSF concentrations start to plateau once blood concentrations surpass 45 μmol/L (Paraskevas et al., 1997). Interestingly, studies have demonstrated a higher CSF-to-plasma ratio in patients with Alzheimer’s disease and major depression as compared to healthy controls (Bowman et al., 2009; Hashimoto et al., 2017). This may reflect an increased utilization of ascorbate by the stressed brain, leading to lower plasma levels (Reiber et al., 1993; Bowman et al., 2009; Hashimoto et al., 2017), or the increased release of ascorbate into extracellular neuronal spaces (Ghasemzedah et al., 1991). One study (Barabás et al., 1995) demonstrated the ratio of dehydroascorbic acid to ascorbate to be higher in CSF than within blood plasma in patients with senile dementia, suggestive of the increased oxidation of ascorbate in the CNS.

### Plasma Vitamin C and Cognitive Function

Given the extensive literature on the biological effects of vitamin C on the CNS, several different human clinical trials have assessed whether plasma vitamin C concentrations affect cognitive function. A meta-analysis of prospective cohort studies showed that the relative risk for dementia was significantly decreased with a higher intake of vitamin C [RR: 0.89 (95% CI: 0.74, 1.06); *p* = 0.192; Cao et al., 2016]. Another meta-analysis assessing plasma vitamin C concentrations in patients with Alzheimer’s disease showed significantly lower values of plasma-based vitamin C (−33%) than those of healthy controls (da Silva et al., 2014).

Based on 50 studies, our recent systematic review examined the link between plasma vitamin C concentrations and cognitive function (Travica et al., 2017). Results revealed higher mean plasma vitamin C concentrations in cognitively intact cohorts compared to those cohorts that were cognitively impaired (Travica et al., 2017). A further qualitative assessment revealed a potential association between plasma vitamin C concentrations and cognition in cognitively intact participants with the need for further research that utilized plasma vitamin C concentrations and sensitive cognitive assessments that would be suitable for cognitively intact adults.

In our recent cross-sectional study, cognitively intact participants presenting with adequate plasma vitamin C levels (≥28 μmol/L), as compared with those with deficient levels (<28 μmol/L), demonstrated significantly better performance on age-sensitive cognitive assessments involving immediate and delayed recall, attention, focus and recognition (Travica et al., 2019). Additionally, a recent randomized controlled trial (Denniss et al., 2019) assessed the effect of a multivitamin/mineral supplement with vitamin C on cognitive function over 8 weeks, in middle-aged adults that revealed significant improvements in verbal and visual memory, visual-motor performance and processing speed. Nonetheless, larger-scale studies, particularly randomized controlled trials, would help reinforce the link between plasma vitamin C and cognition.

## Gender Regulation

Plasma vitamin C variability between males and females has been explored by animal and human studies. Additionally, preliminary studies have examined gender differences in brain ascorbate content and potential factors involved in changing the vitamin distribution and utilization in the brain between genders. A handful of clinical studies have explored a potential interaction between age, plasma vitamin C, and cognitive function.

### Plasma Vitamin C Gender Dimorphisms

A summary of human clinical studies comparing plasma vitamin C concentrations between gender groups is displayed in Table 1. Epidemiological studies have frequently shown higher vitamin C serum/plasma concentrations in women than in men (Birlouez-Aragon et al., 2001; Hampl et al., 2004; Schleicher et al., 2009). Furthermore, with equal vitamin C intake, women achieve higher serum/plasma concentrations than men (Levine et al., 2001; Schleicher et al., 2009). The recent CHALICE study (*n* = 389) demonstrated substantially lower mean plasma vitamin C concentrations in men than in women (*M* = 47.40 μmol/L vs. 40.60 μmol/L, *p* = 0.005) with significantly higher rates of deficiency in men (Pearson et al., 2017). Male gender has also been identified as a risk factor for vitamin C deficiency in hospitalized patients (Fain et al., 2003).

**Table 1:** Summary of human clinical studies comparing plasma vitamin C concentrations between gender groups.

Study/Year	Sample size	Age group	Health status	Result
Birlouez-Aragon et al. (2001)	2,584	>60 years	Healthy	Males 31.6 μmol/L vs. females 40.3 μmol/L, *p* = 0.001
Hampl et al. (2004)	15,769	12–74 years	Healthy	Females exhibited a higher mean serum vitamin C concentration at every age group.
Schleicher et al. (2009)	7,277	>6 years	Healthy	Females reported higher mean serum vitamin C concentration at every age group, particularly in those of 60 years.
Pearson et al. (2017)	404	50 years	Healthy	45.1% of females and 28.7% of males presented with adequate vitamin C
Ness et al. (1996)	1,018	40–59 years	Healthy	Fasting plasma vitamin C levels were significantly higher in women
Fain et al. (2003)	184	>18 years	Hospitalized	Hospitalized males presented with significantly lower mean serum vitamin C concentrations.
Travica et al. (2019)	80	>18 years	Healthy	Females slightly higher concentrations (*p* = 0.46), adequate plasma levels = 47 females (70%) vs. 20 males (30%)

Based on our recent cross-sectional study (Travica et al., 2019) which assessed plasma vitamin C concentrations and cognitive function, a retrospective pilot analysis revealed a marginal, non-significant difference in mean plasma vitamin C levels between genders (males 44.46 μmol/L ± 4.43, females 48.45 μmol/L ± 3.13, *p* = 0.46; Travica et al., 2020). The percentage of males presenting with deficient plasma vitamin C levels (26%) was higher than the deficiency rates of female participants (11%). Those presenting with adequate levels comprised predominantly of females, consisting of 47 females (70%) and 20 males (30%). Additional analyses revealed that the proportion of females with adequate plasma vitamin C levels was higher than males (*p* = 0.001).

Overall, no conclusive gender-related mechanistic differences in the pharmacokinetics of vitamin C have been observed in humans (Counsell and Hornig, 1981; Garry et al., 1982; Blanchard, 1991; Bachar et al., 2016). Although higher urinary excretion of vitamin C has been demonstrated in male rats (Kuo et al., 2004), the expression of vitamin C transporters localized in the proximal tubule brush-border exhibited no differences in their expression between gender groups (Kuo et al., 2004). The tubular maximum reabsorptions and renal thresholds for ascorbic acid are lower in females (86 μmol/L vs. 71 μmol/L; Bachar et al., 2016), suggesting that plasma differences are not explained by differences in the renal handling of ascorbic acid (Oreopoulos et al., 1993).

One proposed mechanism may be variability in the section of cortisol between males and females, with various studies demonstrating higher salivary and plasma cortisol levels (Van Cauter et al., 1996; Seeman et al., 2001). There appeared to be a strong inverse correlation between the ability of an animal to endogenously produce vitamin C and the cortisol response (Hooper et al., 2019). This is observed in guinea pigs that are made deficient in vitamin C hyper-secrete cortisol (Enwonwu et al., 1995) and vitamin C supplementation reducing cortisol levels (Brody et al., 2002). However, a study is yet to directly explore the link between cortisol, and gender plasma vitamin C.

A handful of studies have explored the effects of sex hormones on plasma vitamin C concentrations. Some of the first evidence to postulate a link between hormones and plasma vitamin C came from a rat model which reported that adrenalectomy decreases ascorbic acid and dehydroascorbic acid in the rat liver, the organ primarily responsible for the synthesis and secretion of ascorbate into plasma (Nathani et al., 1971). This investigation was indicative of the importance that adrenal corticoids play in ascorbic acid metabolism. The same authors indicated that mineralocorticoids, such as aldosterone, can influence the metabolism of liver ascorbic acid by reducing the activities of ascorbate degrading enzymes, with its insufficiency possibly being related to elevated ascorbic liver catabolism in males (Nathani and Nath, 1972) and consequently, lower plasma concentrations. Castration in rats was found to lead to a decrease in the activities of biosynthetic enzymes within the liver of ascorbic acid. Additionally, testosterone was also found to restore the increased activity of both renal and hepatic dehydroascorbate following castration (Khandwekar et al., 1974).

One study using female guinea pigs has shown estinyl and progestogen containing contraceptives to reduce circulating ascorbate concentrations (Basu et al., 1974) and estinyl alone to reduce plasma and liver ascorbate concentrations (Basu et al., 1986). The authors hypothesized that these results may be due to these hormones increasing ascorbic acid oxidation and impairing its gastrointestinal absorption. These older studies, primarily restricted to animal studies, demonstrate a potential involvement of hormones in affecting circulating plasma vitamin C concentrations and may potentially play a role in the observed differences in plasma vitamin C concentrations between males and females.

One recent study aimed to assess the effects of gender on serum ascorbate circadian rhythms in humans by collecting serum ascorbate from 162 participants every 6 h for 24 h (Singh et al., 2018). Circadian rhythm characteristics including rhythm-adjusted mean (MESOR) and circadian acrophase (timing of overall high ascorbate values recurring each day) were compared between males and females. However, no gender differences were overserved in these serum circadian rhythm characteristics (Singh et al., 2018).

Recent studies indicate that body weight is considered to be an essential factor for gender-related differences in the pharmacokinetics of vitamin C [German Nutrition Society (DGE) (2015)]. A recent study demonstrated/showed that a higher absolute fat-free mass (FFM) to muscle mass ratio, and thus a higher distribution volume of vitamin C, contributes to lower vitamin C plasma concentrations in men than women (Jungert and Neuhäuser-Berthold, 2015). However, the observed depleted vitamin C levels in obesity are associated with indirect risk factors such as ROS, reduced cardiovascular health, and poor nutrition (Fernández-Sánchez et al., 2011).

Possible lifestyle differences between gender groups may also play a role. Some of these include higher levels of physical activity, alcohol consumption, and smoking amongst males and higher intake of vitamin C containing foods and supplements amongst females (Galan et al., 2005; Jungert and Neuhäuser-Berthold, 2015). Excessive alcohol consumption and smoking have been systematically identified as risk factors for vitamin C deficiency (Lim et al., 2018), leading to the decreased consumption of vitamin C containing foods and increased oxidative stress which biochemically depletes plasma vitamin C concentrations (Schectman et al., 1989). Physical activity, including exercise, has been shown to cause a decline in plasma vitamin C below pre-activity levels during the days following prolonged physical activity (Peake, 2003). This may be a result of physical activity-induced oxidative stress.

Major studies have estimated that only 21–37% of men and 29–45% of women aged 65 and older achieve the recommended fruit and vegetable intake per day (Drewnowski and Shultz, 2001). These differences suggest that increased ROS leads to possible vitamin C utilization in males compared to females. As a consequence, studies have recommended higher daily intakes of vitamin C for males [Levine et al., 2001; German Nutrition Society (DGE), 2015].

### Vitamin C Brain Tissue Gender Dimorphisms

Predominantly animal studies have investigated whether there may be a presence of gender-related differences of vitamin C concentrations in brain tissue. Several studies have revealed female rats have lower basal brain ascorbate concentrations compared to males (Ferris et al., 1995; Kume-Kick et al., 1996).

One of these rat models demonstrated that although male rats had higher cortical vitamin C concentrations (by 7–10%), they demonstrated a greater cerebral vitamin C loss in response to stress and ROS generated during ischemia than females (Ferris et al., 1995).

Male mice with induced Huntington disease (HD) exhibited a 20–40% decrease in striatal ascorbate during behavioral activation that was not observed in females (Dorner et al., 2007). This may have been attributed to higher hypokinesia and a higher susceptibility to oxidative stress within the striatum in males. The authors further postulated that gonadal hormones such as estrogen may contribute to the differences and play a role in modulating striatal ascorbate release. A suggested mechanism is that estrogen could have been involved in the inhibition of striatal oxidative stress associated with HD and (Dorner et al., 2007) preventing glutamate excitotoxicity by direct inhibition of N methyl D aspartate (NMDA) receptors (Weaver et al., 1997).

One study revealed higher basal brain ascorbate concentrations in male rats. However, following gonadectomy, it appeared that loss of the female sex hormones caused a significant ascorbate increase in brain tissue (specifically the hippocampus and cerebellum), comparable to levels in males (Kume-Kick et al., 1996), which was indicative of increased oxidative stress. Moreover, following the induction of oxidative stress through ischemia, female rats who did not undergo gonadectomy experienced three times less brain ascorbate loss than males, whereas increased ascorbate loss was seen in all of these brain regions in females who had undergone the gonadectomy (Kume-Kick et al., 1996). However, only a small subgroup of those who had undergone the gonadectomy was also subjected to ischemia.

The same authors conducted another study that established that in rats, the gender difference in brain ascorbate content did not appear until puberty when sex hormones had reached adult levels (Kume-Kick and Rice, 1998). Furthermore, results demonstrated that changes in sex hormone levels in adult rats can have regionally selective effects on brain ascorbate regulation. This was exemplified by an ascorbate content increase in the hippocampus following ovariectomy in the adult female hippocampus (Kume-Kick and Rice, 1998). Supplementation with 17β-estradiol (E2) treatment following ovariectomy was shown to reduce the ascorbate loss within the hippocampus. The study further revealed that estrogen treatment after ovariectomy prevented enhanced ischemia-induced ascorbate loss, particularly within the hippocampus.

A summary of the studies comparing the distribution of vitamin C in the brain between gender groups is displayed in Table 2.

**Table 2:** Summary of pre-clinical animal studies comparing vitamin C brain distribution between gender groups.

Study/Year	Health status/condition	Brian region differences	Result
Ferris et al. (1995)	Ischemia	The prefrontal cortex, hippocampus, cerebellum	Female rats had lower basal brain ascorbate concentrations. Cerebellum and hippocampus concentrations only fell in males following ischemia.
Kume-Kick et al. (1996)	Ischemia/ gonadectomy	Frontal cortex, hippocampus, cerebellum	Ischemia-induced losses in the brain ascorbate greater in males than females. After gonadectomy, increased ascorbate loss was seen in all-female brain regions.
Kuo et al. (1979)	Ischemia/ gonadectomy	Frontal cortex, hippocampus, cerebellum	Gender differences in brain ascorbate contentwere absent before puberty and persisted only in cortex in aging rats. Estradiol replacement. Ovariectomy prevented enhanced ischemia-induced ascorbate loss in the female hippocampus.
Dorner et al. (2007)	Induced Huntington’s disease	Striatum	Male mice decreased in striatal ascorbate during behavioral activation.
Kuo et al. (2004)	Not specified	Not specified	The male mouse brain exhibited more ascorbate vitamin C receptors than the female brain.
Kume-Kick and Rice (1998)	Ischemia/ gonadectomy	Frontal cortex, hippocampus, cerebellum	Gender differences in brain ascorbate content were absent before puberty and persisted only in cortex in aging rats. Estradiol replacement. Ovariectomy prevented enhanced ischemia-induced ascorbate loss in female hippocampus.

#### Potential Mechanisms Associated With Brain Ascorbate Gender Differences

Several studies have not directly assessed gender brain tissue ascorbate concentrations; however, their results may explain gender differences in the way ascorbic acid is absorbed, distributed, and regulated throughout the CNS which may ultimately affect cognitive function. These results may form the basis for future studies exploring the possible mechanisms associated with gender-related brain vitamin C differences.

Previous reports have indicated increased antioxidant enzymes in the male gender, which may be associated with greater basal oxidative stress and enhanced brain ascorbate levels to compensate for oxidative stress. In the prefrontal cortex, hippocampus and cerebellum, males have 13–50% times greater superoxide dismutase (SOD; Carrillo et al., 1992), with SOD constituting a very important antioxidant defense against oxidative stress in the body (Landis and Tower, 2005).

Recent studies report that female mitochondria generate half the amount of hydrogen peroxide compared to mitochondria of males and contain higher levels of antioxidant enzymes and compounds (Ostan et al., 2016). Moreover, GSH peroxidase activity is lower in males, leading to a greater generation of hydrogen peroxide and ROS in the brain (Ferris et al., 1995). Additionally, male mice seem to experience a more dramatic age-associated decline in GSH content than female mice in many tissues, including the brain (Wang et al., 2003). The lowered defenses against ROS imply that the male brain may require more vitamin C for neuroprotective purposes.

Also linked with oxidative stress is mitochondrial function, which has recently shown gender variations in the human brain. Using H-magnetic resonance spectroscopy, a recent pilot study revealed significantly higher mitochondrial content within the female brain and consequently higher concentrations of the metabolite N-acetyl aspartate (NAA; Silaidos et al., 2018). This is in line with a study conducted by Rutkai et al. (2015) which observed higher mitochondrial respiration in freshly harvested cerebral arteries from adult female rats compared to males. Based on these findings, it has been postulated that mitochondrial dysfunction, as a result of higher levels of oxidative stress, may account for the lower apparent NAA levels in males. Mitochondria from females exhibit better coping with stressful conditions and are relatively resilient to DNA damage and mutations (Demarest and McCarthy, 2015). These differences in CNS mitochondrial function may influence the diverse regulation of the brain ascorbate between genders.

Also, the aging male brain may be more vulnerable to experiencing excitotoxicity as a result of higher extracellular glutamate levels (Sailasuta et al., 2008), which can result in a higher efflux of ascorbic acid into the extracellular space as a means of preventing neuronal loss.

Differences in the distribution of brain vitamin C receptors may result in absorption differences between males and females. Vitamin C is primarily transported into the CSF and neurons through sodium-dependent vitamin C transporters 2 (SVCT2; Portugal et al., 2017). Although a study revealed comparable numbers in SVCT2 expression between *young* female and male mice (Kuo et al., 2004), the expression of SVCT2 receptors is mediated by inflammation and oxidative stress. Increases in extracellular ascorbic acid induce increases in SVCT2 on neuronal cell surfaces, leading to increased intracellular uptake of ascorbic acid (Acuña et al., 2013) which may explain the higher concentrations in males as a result of increased oxidative stress.

Furthermore, following a pro-inflammatory challenge, microglia, defined as the brain’s resident macrophages, reduce the expression of their SVCT2 receptors (Portugal et al., 2017). As a result, reduced ascorbate uptake in microglia leads to ineffective inhibition of NF-κB and the induction of pro-inflammatory mediators such as TNFα, IL-1β, IL-6, and iNOS. This highlights the importance of intracellular vitamin C transport and needs by brain cells, regardless of the amount that is present within the CSF. To date, there is a lack of research comparing the CSF to plasma vitamin C ratios between healthy or clinically diagnosed gender groups.

Moreover, there appears to be gender differences in vitamin C brain turnover as a result of hormonal factors. The studies mentioned earlier conducted by Kume-Kick et al. (1996) and Kume-Kick and Rice (1998) indicated that hormones such as estrogen can modulate not only brain ascorbate levels but also brain redox status in a region-specific manner. This was exemplified by gonadectomy particularly reducing levels in the hippocampus in female rats and gonadectomy leading to an even wider spread of ascorbate loss during oxidative stress than non-gonadectomy in females. Taken together, these findings support the hypothesis that sex hormones influence ascorbate levels and overall redox balance in the female brain differently to the male brain.

Other studies have investigated the effects of the hormones on a more molecular level within the brain. Ovariectomy decreased brain mitochondrial oxidative phosphorylation and increased oxidative stress (Razmara et al., 2007) while Gaignard et al. (2015) demonstrated a sex difference in brain mitochondrial respiration and oxidative stress that is suppressed with ovariectomy.

Further supporting the potential role of hormones in ascorbic brain regulation are the results from a study which assessed the role of hormones on the gene expression of choroid plexus (CP) circadian rhythms, which were compared between females and males using cultured CP cells (Quintela et al., 2015). The preliminary results demonstrated gender-associated differences in the circadian expression of CP clock genes. Results further suggested that gender hormones can regulate circadian rhythmicity in the choroid plexus and potentially affect the regulation and homeostasis of ascorbate concentrations within the brain. However, the way these differences influence the regulation of ascorbate CSF directly has not been examined.

An additional study conducted by the same authors addressed differences between males and females on the effects of sex hormones in the CSF production and composition (Quintela et al., 2016) by identifying sex-related differences in the choroid plexus transcriptome and CSF proteome (Quintela et al., 2016). There were five down-regulated proteins in the CSF of male rats compared to females. One of these proteins (APOA1) was the main protein component of high-density lipoprotein-like particles that play an important role in lipid metabolism in CSF. APOA I has antioxidant and anti-inflammatory properties and plays a role in amyloid β (Aβ) aggregation. These potentially lower antioxidant and higher inflammatory properties may affect ascorbate regulation within the CSF of males. Interestingly, orchidectomy in males up regulated a majority of these proteins whereas ovariectomy did not induce changes in the CSF proteome.

Growing evidence suggests that telomere length is a marker of biological aging, with telomere shortening potentially being linked to age-related disease and longevity (Codd et al., 2013). Recently, it has been proposed that shorter leukocyte telomere length may be associated with increased brain aging, marked by structural brain changes in cortical thickness (Puhlmann et al., 2019) and the volume of multiple brain regions (Jacobs et al., 2014), possibly predicting preclinical cognitive decline (Rask et al., 2016). Consistent findings have put forward that females have longer average telomere lengths than males (Gardner et al., 2014). Moreover, gender differences in plasma vitamin C concentrations and oxidative stress may play a role in the gender-related telomere differences, with studies postulating a link between telomere length and vitamin C intake in addition to plasma vitamin C concentrations (Sen et al., 2014; Mazidi et al., 2017). Taken together, gender-associated variances in brain aging may be affected by differences in telomere length which are influenced by gender-related plasma vitamin C and oxidative stress. The variability in brain aging could consequently contribute to gender-associated variance in brain vitamin C regulation and cognitive function.

Additionally, structural brain differences between genders may contribute to differences in brain ascorbate distribution. One study assessed the neuronal density in four areas of the hippocampus, and entorhinal and frontal cortices to analyze the possible gender influence during normal aging (Martínez-Pinilla et al., 2016). Results observed a higher neuronal density of certain hippocampal areas of the non-pathological brains of young men compared to women. Further studies on the cerebral cortex show that men have 15% more cortical neurons and 13% greater total neuronal density than women. The largest single-sample study of structural and functional sex differences in the human brain revealed that males had substantially larger brain volumes and surface areas, whereas females had thicker cortices (Ritchie et al., 2018). Previously it has been suggested that ascorbate content increased linearly with increasing neuron density (Rice and Russo-Menna, 1998). Given the larger volumes and neuronal density, this may be suggestive that in males, ascorbate needs to be distributed across a larger brain volume and possibly requires more ascorbate to exert optimal function. This is also in line with lower plasma levels in males being the result of a larger body mass distribution.

Due to a larger abundance of cell bodies, ascorbate concentrations are higher in the gray matter than in white matter (Rice, 2000). Differences in the distribution of gray matter between males and females have been identified. These differences are thought to be due to the combination of the neuroprotective effect of estrogen (which is more prominent in females) tending to decrease the loss of gray matter (Taki et al., 2011), particularly within the hippocampus (Österlund et al., 1999). An MRI study revealed that the cerebellar gray matter volume in males was significantly larger in the anterior and middle posterior lobes (Taki et al., 2011) while others have recently detected more gray matter volume within subcortical temporal structures in men, which included the putamen, anterior cerebellum and premotor cortex (Lotze et al., 2019). These results could potentially account for differences in the regional specific distribution of ascorbic acid throughout the brain between genders.

Although plasma ascorbate is not primarily transported through the blood-brain barrier (BBB), the integrity of this barrier may impact the stability of ascorbate concentrations within CSF (Bowman et al., 2009), with increased permeability showing reductions in the ratio of CSF to plasma ascorbic acid. This may result from elevated oxidative stress and the diffusion of ascorbic acid out of the CNS according to its concentration gradient (Bowman et al., 2009). Recently, a study examining the impact of gender on the blood-CSF barrier discovered a higher rate of a dysfunctional barrier in males than in females (Castellazzi et al., 2020). Taken together, these findings may explain the involvement of BBB integrity in differences within CSF ascorbic acid concentrations between genders. However, this research was limited to those diagnosed with neurodegenerative conditions and needs to be confirmed using healthy cohorts.

The relationship between sex and cerebral blood flow (CBF) were studied extensively in the past, and most authors have reported higher CBF in women than in men (Aanerud et al., 2017). This may be indicative of more efficient delivery of blood ascorbate into the choroid plexus in females. Additionally, differences in the density of the CSF have been observed between males and females which could affect the distribution of ascorbate in this fluid between genders (Lui et al., 1998).

### Interaction Between Gender, Plasma Vitamin C and Cognition—Human Clinical Studies

A study conducted on a large cohort of elderly Korean participants reported a significant relationship between vitamin C consumption and Mini-Mental State Examination scores in men, but not in women (Lee et al., 2001). Another study revealed that men who reported low MMSE scores consumed less vitamin C than females with low MMSE scores, however, little variability in vitamin C consumption was observed between both gender groups in those who demonstrated high MMSE scores (Ortega et al., 1997).

Sato et al. (2006) assessed the interaction between gender and plasma vitamin C concentrations on cognitive function using a cognitively intact sample. An analysis by gender was examined in this study, showing that higher plasma concentrations were associated with higher MMSE scores for men, albeit non-significantly. Age was associated with lower MMSE scores in men but not in women, despite a similar range of plasma vitamin C concentrations and MMSE scores between genders. Digit Symbol Substitution Test scores improved with increasing plasma concentrations for both gender groups. The authors concluded that more sensitive cognitive assessments were required to determine true associations in cognitively intact cohorts.

Previous investigations that assessed vitamin C intake or plasma concentrations controlled for gender as a potential confounding factor rather than considering a potential interaction between gender, plasma vitamin C, and cognitive function (Gale et al., 1996; Perrig et al., 1997).

We conducted a retrospective, pilot analysis on data gathered from our published cross-sectional study (Travica et al., 2019) on healthy adults (*n* = 80, female = 52, male = 28, 24–96 years), primarily consisting of blood plasma vitamin C concentrations as well as cognitive function. Cognitive assessments included the Swinburne University Computerized Cognitive Assessment Battery (SUCCAB; Pipingas et al., 2010) which assessed reaction time in milliseconds for each of the battery’s eight tasks as well as a percentage relating to performance accuracy. Two pen and paper tests, which included the Symbol Digits Modalities Test (SDMT) and the Hopkins Verbal Learning Test-Revised (HVLT-R) were also used to assess cognition.

After adjusting for several potential covariates such as age, the number of prescribed medications and dose of self-reported vitamin C supplementation, results indicated a significant interaction (*p* < 0.001) between plasma vitamin C and gender on cognitive function, on both the computerized and pen and paper assessments (Travica et al., 2020). Both males and females with adequate vitamin C concentrations outperformed those with inadequate concentrations. Females with adequate vitamin C levels also exhibited higher performance than males on tasks involving recall, recognition, attention, and focus. Additionally, the performance of males with inadequate plasma vitamin C was poorer on tasks involving components of memory (short/delayed), inhibition, and visual perception, whereas females presenting with a vitamin C deficiency were more compromised on tasks involving psychomotor performance/motor speed.

## Age Modulation

Plasma vitamin C variability during aging has been explored by various animal and human studies. Additionally, studies have aimed to examine changes in ascorbate content within the aging brain and potential factors involved in changing the vitamin distribution and utilization in the brain during aging. A handful of clinical studies have explored a potential interaction between age, plasma vitamin C, and cognitive function.

### Plasma Vitamin C and Age

Despite the possible modulatory action of gender, the literature suggests that age may also affect plasma vitamin C and cognition link. It has been established that plasma and erythrocyte ascorbate concentrations significantly decline with age (Rizvi et al., 2009; Bachar et al., 2016). This has been supported by several population-based studies in which those aged above 60 years were more prone to present with plasma vitamin C deficiencies (Bates et al., 1999). Similarly, 40% of elderly individuals residing in nursing homes or residential homes (*n* = 423) have been shown to display deficient plasma vitamin C levels, with a mean level of 24.4 μmol/L (Bates et al., 1999).

Based on a population study, the percentages of 12–17-year-old males and females who were vitamin C deficient were low (5–6%) relative to other groups (Hampl et al., 2004). Also, a meta-analysis of 36 publications examining the relationship between vitamin C intake and plasma concentrations of vitamin C concluded that older adults (aged 60–96 years) have considerably lower plasma levels of vitamin C following a certain intake of vitamin C compared with younger individuals (aged 15–65 years; Brubacher et al., 2000), suggesting a change in absorption and pharmacokinetics. Several explanations have been postulated for the aged-associated plasma vitamin C depletion. Multiple mechanisms could be involved including increased usage, accelerated turnover, decreased absorption/reabsorption, and reduced cellular uptake.

Pharmacokinetic studies in older adults have not yet been conducted, but evidence suggests that the efficiency of one of the molecular mechanisms for the cellular uptake of vitamin C declines with age (Michels et al., 2003). When incubated with 100 μM of ascorbate, cells from old as compared to young rats showed a 66% decline in both the rate of ascorbate transport and the steady-state intracellular levels, with an SVCT1 decline of 45% with age (Michels et al., 2003).

Higher blood glucose levels, that are commonly observed in the elderly (Chia et al., 2018), may be linked with compromised recycling of DHAA due to its transporters being competitively inhibited by glucose, with excess glucose in plasma or intestine blocking the receptor-binding site and decreasing GLUT-facilitated DHAA transport (Wilson, 2003). Additionally, systemic inflammation, which is more pronounced in the elderly (Sanada et al., 2018) affects gut permeability. Prolonged inflammation and dysbiosis increase the translocation of lipopolysaccharide gram-negative bacteria to potentiate additional inflammation. Lipopolysaccharides inhibit the absorption of ascorbate by decreasing sodium-dependent vitamin C transporters (Subramanian et al., 2018a). Others have postulated that tumor necrosis factor-α (NF-κB) inhibits intestinal ascorbic acid uptake in both *in vitro* and *in vivo* systems, and this inhibitory effect is partially mediated by the transcription of the sodium-dependent vitamin C transporter-1 gene *via* the NF-κB pathway (Subramanian et al., 2018b).

Vitamin C is a strong reducing agent (i.e., an electron donor), both *in vivo* and *in vitro*, and the lower levels of blood vitamin C reported among the elderly may be due to a higher turnover of vitamin C which is explained by increased oxidative stress with increasing age (Institute of Medicine Panel on Dietary and Related, 2000). Several key biochemical functions rely on ascorbate donating electrons, a process that is amplified during the aging process which results in reduced plasma ascorbate levels.

Additionally, the ascorbate decline may be due to reduced ascorbate recycling (Bachar et al., 2016) as a result of aged-associated glutathione depletion. To compensate for the aged-associated increases in oxidative stress, a compensatory mechanism has been established whereby increased erythrocyte ascorbate free radical (AFR) reductase is involved in the reduction of AFR to ascorbate in the plasma (Rizvi et al., 2009). Also, several age-associated hormonal changes that effect corticosteroids and estrogen may affect circulating ascorbate levels (Basu, 1986). Contrary to the observed plasma depletion during aging, seniors are more likely than younger individuals to purchase and use nutrient supplements, particularly vitamin C (Loria et al., 1998; Vitolins et al., 2000). Although, on average, older adults eat more servings of fruits and vegetables, which might be nutritionally necessary given the change in metabolic processes that occur in old age (Lichtenstein et al., 2008).

The intake of fruits and vegetables in the elderly fails to reach recommended levels and most nutritious and health-promoting foods have been reported to be under-consumed (Nicklett and Kadell, 2013). One study found that 33% of community-dwelling British adults 65 years or older (*n* = 1,310) consumed less than the recommended intake for vitamin C (Bates, 1993). Additionally, the vitamin C content present in foods consumed by the elderly may be affected by storage, heating, freezing, and food preparation techniques, particularly those in hospitals and nursing homes (Armstrong et al., 2019). A recent systematic review aimed to determine the extent that vitamin C is lost from food, secondary to food cooking methods used in hospitals and care facilities. Results identified significant vitamin C losses between preparation and service resulting from food cooking methods in hospitals and care facilities (Armstrong et al., 2019). Older adults’ eating habits are also heterogeneous, and the determinants of fruit and vegetable intake among older adults are complex. With the population aging, the number and intensity of barriers in accessing and consuming fruits and vegetable increase (Nicklett and Kadell, 2013).

Older adults are at heightened risk of functional limitations, disability, and chronic disease onset and complications (Hampl et al., 2004). Being affected by these conditions makes accessibility, preparation, and consumption of these important nutrients problematic. Old age is often accompanied by changes in appetite, declines in chewing efficiency, and compromised oral health which could reduce fruit and vegetable intake (Whitelock and Ensaff, 2018).

In both genders, older age was significantly associated with higher levels of salivary cortisol measures, most consistently with evening cortisol (Larsson et al., 2009). The negative feedback regulation of the HPA-axis seems to become impaired in older subjects (Wilkinson et al., 1997). As already mentioned, there is a likely inverse correlation between the ability of an animal to endogenously produce vitamin C and the cortisol response (Hooper et al., 2019).

With increasing age, circadian rhythms tend to fluctuate more, which may influence plasma ascorbate levels. One recent study specifically assessed how aging affects serum ascorbate circadian rhythms by assessing the ascorbate rhythm-adjusted mean (MESOR) and circadian amplitude (Singh et al., 2018). The MESOR increased significantly until 46.5 years and the circadian amplitude also started to decrease around 42 years of age. There was a progressive decline of the circadian acrophase (timing of overall high values recurring each day) of ascorbic acid with increasing age.

### Vitamin C Variations in Brain Tissue Between Age Groups

Studies have predominantly focused on the effects of aging on plasma vitamin C concentrations, with a handful of studies aiming to raise insights into the effects of aging on CNS ascorbate concentrations. These studies have consistently demonstrated an age-related decrease of ascorbate levels in the brain, especially within the cerebral cortex, pituitary gland, and hippocampus (Schaus, 1957; Siqueira et al., 2011). One of these studies demonstrated that the ascorbate level in the cerebral cortex is decreased 77% from individuals at age 80 and older, compared to individuals at age 50 and younger. Vitamin C levels in tissues, such as the brain and muscle are reduced with age to as little as 25% of those found in children (Lewin, 1976).

Significant differences in CSF ascorbate content were exhibited by premature and term babies, with premature babies having up to 16-times higher values of vitamin C in CSF than in plasma, whereas vitamin C concentrations in CSF of controls were on average 2.5–3 times higher in CSF than in plasma (Heinz-Erian et al., 1985). Furthermore, CSF vitamin C was 4–5 times higher in premature babies than in school children. These results, particularly those arising from premature babies, postulate the body’s utilization of ascorbate for important biological central nervous system functions, such as neurodevelopment and neuroprotection (Heinz-Erian et al., 1985).

A study that conducted 67 human autopsies revealed a large depletion in brain vitamin C concentration with age, with higher concentrations found in younger subjects (<10 years) compared to those over 10 years of age (Yavorsky et al., 1934). Similarly, research has demonstrated a gradual decline in vitamin C concentration within CSF from the age of 10 years onwards (Stephenson et al., 1941).

More recent research has used human brain spectroscopic MRI imaging to compare ascorbate, glutathione, and lactate concentrations in the brain on 22 young (20 years) vs. 22 normally aging (76.6 years) participants (Emir et al., 2011). The study reported decreased glutathione and increased lactate with age, indicating oxidative damage, but no change in ascorbate between the groups. However, the sample consisted of a cohort that was consuming low amounts of fruits and vegetables and not supplementing vitamin C before assessment. Additionally, the sample was on a controlled diet (food containing 30 mg/1,000 kcal of vitamin C) during the trial as a means of providing a recommended daily intake to participants, thus, potential group differences were minimized, and circulating and brain ascorbate levels may still have been sub-optimal in all subjects.

Animal studies have directly assessed the effects of aging on brain ascorbate concentrations. One study revealed decreased ascorbate uptake in hippocampal slices from old-aged rats, with the decline in uptake being involved in greater susceptibility to oxidative damage with advancing age (Siqueira et al., 2011). A previous study by the same authors reported that the observed compromises in hippocampal ascorbate uptake may be the result of decreases in total reactive antioxidant potential and total antioxidant reactivity levels, with both indexes almost 30% lower in aged rats compared to younger ones (Siqueira et al., 2005).

Based on an animal model, aging was associated with a significant reduction in cerebral α-tocopherol, ascorbate and glutathione contents (Sahoo and Chainy, 1997) and the activity of enzymes such as SOD catalase, glutathione peroxidase and glutathione reductase (Sahoo and Chainy, 1997). The results suggest that through aging, increased free radicals could be the causative agents of reductions in these enzymes which propagates into diminished ascorbate recycling capacity. This trend has been reiterated (Lykkesfeldt and Moos, 2005) in a study which found plasma vitamin C status of the young guinea pigs (also unable to synthesize vitamin C) to significantly decline to that of the old animals within 3 months, suggestive of a possible decline in plasma vitamin C status and neuronal tissue earlier than old age.

#### Potential Mechanisms Involved in Age-Associated Brain Vitamin C Variability

Various studies have examined several age-associated factors that may contribute to changes in brain vitamin C absorption, distribution, and utilization during aging. Although these studies did not directly assess brain ascorbate concentrations, they provide an insight into the potential mechanisms influencing brain vitamin C.

Reductions in ascorbic acid and glutathione (GSH) may be the result of age-related variations of total antioxidant defenses in the brain, predisposing structures to oxidative stress, and depleting ascorbate concentrations (Siqueira et al., 2005). A reduction in mitochondrial functions, including the activity of electron transport chain complexes, GSH levels as well as the antioxidant defense enzymes such as SOD, was observed with increasing age (Ighodaro and Akinloye, 2018). Given ascorbate’s substantial involvement in neutralizing ROS, ascorbate would be extensively utilized for neuroprotective purposes within the aging brain, contributing to eventual neural reductions of ascorbate.

CSF turnover can also be disrupted during aging. One recent study demonstrated significantly reduced CSF flow during aging by calculating ventricular and spinal CSF flow in a cohort of elderly participants (Attier-Zmudka et al., 2019) which was associated with cognitive performance. Since CSF is important for transporting ascorbate into the extracellular neuronal space and is the fluid which neurons are exposed to, possibly the impaired CSF flow during aging could be associated with reduced transportation of ascorbic acid into neurons. This may also compromise dehydroascorbic acid recycling within an ECF, alongside the accumulation of metabolites that contribute to added oxidative stress.

Additionally, resting CBF has been shown to significantly slow during aging (Tarumi and Zhang, 2018) reflected by decreased cerebral metabolic rate and cerebrovascular dysfunction. This may affect the amount of ascorbate that crosses the epithelial cells of the choroid plexus and reaches the CSF, also potentially contributing to the accumulation of metabolic products, and contributing to ascorbate loss. Brain areas consisting of a large number of cell bodies, such as gray matter volume, decreases with age in networks containing subcortical structures, sensorimotor structures, and cingulate cortices (Hafkemeijer et al., 2014). The cerebral cortex can also display thinning and reduced cell bodies with age (Salat et al., 2004). It is known that the concentrations of ascorbic acid are most prominent in brain cells containing large numbers of cell bodies and gray matter (Rice, 2000). Taken together, these findings may suggest a reduction in ascorbic acid concentrations in brain tissue due to the loss of gray matter and cell bodies.

Alterations in brain hormonal concentrations throughout the lifespan may also affect brain ascorbate concentrations. The decrease in gonadal hormone production during aging is gradual in men (testosterone), but estrogen levels in women promptly decrease after menopause (Gaignard et al., 2015). As discussed earlier, alterations in hormonal levels may contribute to changes in both plasma concentrations as well as brain ascorbate concentrations. Estrogen plays an important role during adulthood not only in the estrous cycle but also in the brain *via* neuroprotective, neurotrophic, and antioxidant modes of action (Lejri et al., 2018). Menopause in women, which is characterized by significant reductions in brain ascorbate is particularly linked with increased brain oxidative stress which alters the way ascorbate is distributed and utilized in the brain, as observed in female species following gonadectomy (Kume-Kick et al., 1996).

Several studies have assessed the effects of aging on receptors responsible for transporting vitamin C into and throughout the brain. One rat study found that SVCT2 expression followed an inverse relationship with ascorbate levels in the developing brain (Meredith et al., 2011). Particularly within the cortex and cerebellum, ascorbate levels were high throughout the late embryonic stages and early post-natal stages and decreased with age. Conversely, SVCT2 mRNA, and protein levels were low in embryos and increased with age. Results further suggested that even the relatively low levels of SVCT2 are adequate to maintain the high ascorbate levels in cortex and cerebellum during embryogenesis. The authors further suggested that the increase in SVCT2 mRNA and protein during post-natal development could thus be linked to the increase in neurogenesis and increased oxidative stress present after birth and may decrease ascorbate content with age while also up-regulating the SVCT2 receptors (Meredith et al., 2011).

Peripheral blood DHAA crosses the BBB through the glucose receptors—GLUT1. The expression of BBB GLUT1 receptors may be compromised during aging as observed in age-associated cognitions such as Alzheimer’s and diabetes (Shah et al., 2012; Winkler et al., 2015). Given the reduced expression of these receptors, the absorption of DHAA into the CNS may be reduced and restricted from being recycled into ascorbic acid. Furthermore, elevated blood glucose levels, which are commonly observed during aging, may also compete for the absorption of DHAA through GLUT1 (Agus et al., 1997).

As discussed, BBB integrity may play a role in the brain’s ability to maintain ascorbic acid within the CSF (Bowman et al., 2009). Multiple age-related factors may contribute to increasing the permeability of the BBB and potentially reducing CSF ascorbic acid concentrations. These include age-related increases in inflammation, mitochondrial dysfunction, and dysfunction of transporters and receptors (Tiani et al., 2019). Recent studies have demonstrated that BBB permeability is dynamically controlled by circadian rhythms and sleep (Cuddapah et al., 2019). Molecules such as cytokines, hormones, and peptides which affect BBB integrity have been observed to undergo circadian oscillations. Also, sleep promotes the clearance of metabolites along with the BBB, and endocytosis of vital molecules through the BBB. However, these functions may be disrupted as a consequence of aging, as increasing age has been shown to significantly affect circadian sleep and rest-activity rhythms, with estimates of actual sleep time and sleep efficiency decreasing significantly during aging (Huang et al., 2002).

As mentioned, telomere length may have implications on brain aging and cognitive function (Jacobs et al., 2014). It is known that the vulnerability of telomere shortening is heightened in older individuals (Takubo et al., 2002). Given the potential involvement of vitamin C in preserving telomere length (Sen et al., 2014), it can be predicted that the age-associated plasma vitamin C decline may be involved in telomere shortening. Collectively, these findings propose that age-associated telomere shortening, which is potentially linked with plasma vitamin C, may contribute to brain aging and therefore the regulation of vitamin C within the brain.

Another possible explanation for reductions in brain ascorbate concentrations during aging may be the result of a compromised ability of astrocytes to recycle DHAA within the extracellular space. During aging, astrocytes are more vulnerable to being exposed to oxidative stress, affecting astrocyte density and dysfunction (Palmer and Ousman, 2018). Astrocytes that are exposed to oxidative stress factors during aging begin to undergo oxidative stress themselves, experiencing oxidized DNA in their nuclei (Lei et al., 2008). They may also lead to a decline in glutathione concentrations within the astrocyte and astrocytic NADPH as observed during aging (Jiang and Cadenas, 2014). This may ultimately compromise the ability of astrocytes to recycle DHAA given that astrocytes need to reduce DHAA into ascorbic acid, a process that depends on glutathione and NADPH (Li et al., 2001).

### Interaction Between Plasma Vitamin C, Cognition and Age—Human Clinical Trials

Given the postulated changes in vitamin C concentrations within plasma and CNS during aging, preliminary human clinical trials have assessed the interaction between age, vitamin C, and cognition. A comprehensive review of the literature has shown that age-associated neurodegenerative diseases are generally associated with low plasma vitamin C levels and that maintaining healthy vitamin C levels can have a protective function against age-related cognitive decline, including Alzheimer’s (Harrison and May, 2009). Plasma levels of vitamin C are lower in patients with mild cognitive impairment and neurodegenerative diseases including Alzheimer’s and dementia compared to controls (Travica et al., 2017). Such severe cognitive declines that are associated with these clinical conditions may be a reflection of severe vitamin C depletion observed during aging.

Studies that targeted cognitively intact, middle-aged adults revealed mixed results between vitamin C consumption and cognition, but were restricted to assessing dietary intake instead of blood concentrations of vitamin C, and failed to administer cognitive assessments suitable for cognitively intact cohorts (Peacock et al., 2000; Berti et al., 2015; Beydoun et al., 2015). One of these included a cross-sectional study that assessed the dietary intake, by using a 24-h recall diary, on a large cohort with a mean age of 47.5 ± 9.3 years. Results failed to demonstrate an association between self-reported dietary intake of vitamin C and cognitive function on a broad range of cognitive assessments (Beydoun et al., 2015). An additional cross-sectional study assessed the association of dietary and supplemental vitamin C intake with cognitive function in a cohort aged between 48–67 years, revealing no consistent associations between dietary vitamin C intake or supplement use and any of the cognitive tests (Peacock et al., 2000).

Another cross-sectional study assessed dietary vitamin C consumption alongside other antioxidants in a cohort of women with a mean age of 54 ± 12 years and an MMSE score over 27 (Berti et al., 2015). The study also assessed a marker of cerebral glucose metabolism (METglc). A positive association between dietary intake of vitamin C and METglc was overserved, with higher vitamin C consumption being linked to more efficient brain glucose metabolism.

A large prospective cohort study was conducted on a sample between the ages of 45–60 years at baseline (Péneau et al., 2011). A 13 year follow up, which consisted of a 2 monthly food diary assessment, revealed that vitamin C intake was positively associated with verbal memory scores in particular.

On the other hand, studies testing older (>65 years), cognitively intact participants have incorporated plasma vitamin C assessments more readily. These studies consistently displayed a significant link between plasma vitamin C concentrations and a range of cognitive domains, including free recall, recognition, and vocabulary (Goodwin et al., 1983; Perrig et al., 1997; Sato et al., 2006).

Several previous investigations that have assessed a broader range of age groups have statistically controlled for age as a potential covariate (Peacock et al., 2000; Travica et al., 2019). One study conducted by Chaudhari et al. (2016) consisted of a rural cohort with a broad range of ages between 40–96 years. In an analysis that did not control for age as a potential confounder (unadjusted model), self-reported vitamin C supplementation was associated with significantly better immediate memory, visuospatial skills, and global cognitive functioning across the entire cohort. However, participants were not stratified into specific age groups and compared for any variations in vitamin C intake and cognition.

There is a scarcity of research assessing the interaction between age, plasma vitamin C, and cognition. Based on the previous clinical studies that assessed the link between vitamin C concentrations and cognitive function, a majority of studies were conducted on participants over the ages of 65 years, with limited data on middle-aged and younger adult populations. The few studies that did incorporate mid-aged adult groups, relied on cross-sectional designs and failed to assess vitamin C concentrations using blood tests, relying heavily on FFQs alone, which have only demonstrated moderate correlations with blood concentrations (Dehghan et al., 2007).

Furthermore, a majority of these studies failed to administer cognitive assessments suitable for a broad range of age groups, primarily relying on the Mini-Mental State Examination to assess cognitive function (Travica et al., 2017). As a result, it is not clear how cognition in certain age groups, particularly in those under the ages of 65 years. is affected by plasma vitamin C concentrations and whether this varies with older age groups (>60 years). At this point, a high heterogeneity between study designs makes the assessment of a potential interaction between age, plasma vitamin C, and cognition difficult.

## Limitations and Future Directions

The link between plasma vitamin C and cognitive function in cognitively intact participants has recently come to light, with recent clinical investigations suggesting a possible link between plasma vitamin C and cognition. Nonetheless, larger-scale studies, assessing vitamin C using blood samples, suitable cognitive assessments, and even assessing brain tissue concentrations will help further confirm the link between plasma vitamin C and cognition. Additionally, investigations into the link between plasma vitamin C and brain tissue regulation are further needed to help determine how plasma vitamin C concentrations affect brain tissue vitamin C.

Previous research directly exploring vitamin C brain tissue gender dimorphisms and age variability is limited to animal studies, particularly rats who can synthesize vitamin C (unlike humans) and have significantly lower neuron density than humans (Rice and Russo-Menna, 1998). Although these animal models offer preliminary findings which could be potentially generalized to humans, future research should endeavor to incorporate human participants for more conclusive results.

The pharmacokinetics of vitamin C, including the absorption, distribution, metabolism, and elimination are quite complex and involve several different active and passive transport mechanisms (Lindblad et al., 2013), alongside intracellular reduction permitting the recycling of vitamin C within the brain. This may account for the limited available evidence directly assessing brain vitamin C variability between gender and age groups. This opens a scope of research for future investigations which could aim to explore the possibility of variations in these pharmacokinetics between age and gender groups.

Additionally, the proposed mechanisms that potentially relate to differences in plasma and especially brain vitamin C between genders and aging need to be interpreted with caution. Although multiple general differences have been observed in the CNS between gender groups and during aging, these studies have failed to directly assess their effects on vitamin C concentrations within the brain. The proposed mechanisms involved in affecting the brain and plasma vitamin C regulation require further focus, particularly assessing whether these may vary between gender and age groups. Future research could focus on establishing which factors may be linked to brain vitamin C absorption, distribution, and utilization during aging. Furthermore, the distribution of vitamin C within the brain and CNS according to varying plasma vitamin C concentrations between gender and age groups is warranted.

More clinical studies investigating the interaction between age and gender, with plasma vitamin C and cognition are needed. This will clarify whether variations in plasma vitamin C may be linked to gender and aged-associated differences in cognition. To date, a majority of studies were conducted on participants over the ages of 65 years, with limited data on middle-aged and younger adult populations. Future studies should aim to determine which cognitive abilities would be affected differently as a result of the gender vitamin C variation. Our pilot study has briefly examined this by suggesting that certain tasks are affected differently. At this stage, it is unknown whether variations in ascorbate status affect different cognitive tasks diversely between gender and age groups (Travica et al., 2020).

Studies that do take into account gender and age should aim to assess both peripheral blood and brain levels of oxidative stress through SOD, glutathione peroxidase, and malondialdehyde. This would give a clearer indication of any variability between gender and age groups. Assessing DHAA concentrations between genders should be implemented to determine the amount of ascorbic acid that is being oxidized, as well as SVCT2 receptor variations. These studies should also take sex hormones into account and determine whether female samples are post-menopausal, a factor that seemingly affects brain vitamin C concentrations based on animal models. As discussed in our previous work, future human studies should continue to use cognitive measurements suitable for distinguishing subtle differences in cognitively intact samples and cognitive assessments that are suitable for a broad range of age groups.

## Summary and Conclusion

**Figure 2:**
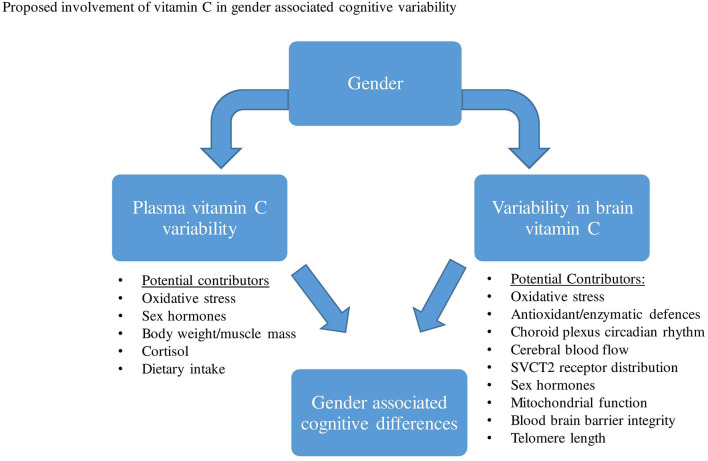
Various factors have been proposed that may contribute to both plasma vitamin C and brain variability between males and females. This variability in plasma and brain vitamin C may then contribute to gender-related cognitive differences.

Thus far, epidemiological studies have frequently shown higher vitamin C serum/plasma concentrations in women than in men. The exact mechanisms responsible for plasma vitamin C differences between males and females have not been confirmed, however, both pharmacokinetic and/or lifestyle variability have been proposed. Based on previous animal investigations that measured brain vitamin C concentrations directly, results demonstrated that the CNS distribution and utilization of ascorbate may vary between gender groups, particularly while females are pre-menopausal. Several proposed mechanisms may account for gender differences in brain vitamin C regulation and utilization, which include differences in brain hormones, oxidative stress, CBF, choroid plexus, SVCT2 distribution, et cetera (Figure 2).

**Figure 3:**
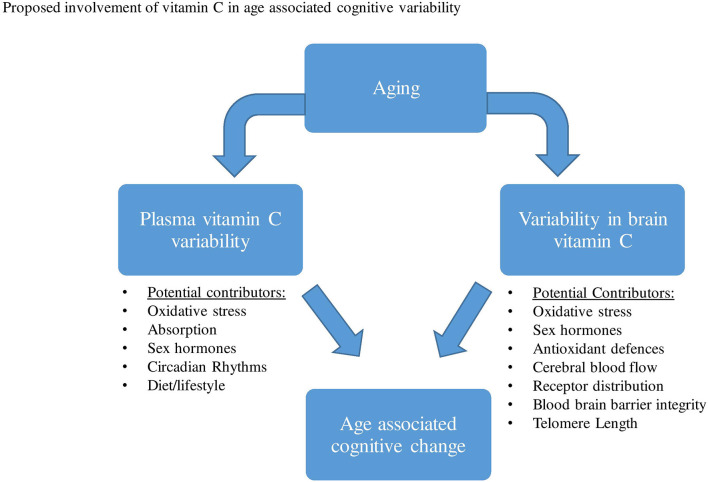
A range of factors has been proposed that may contribute to both plasma vitamin C and brain variability during aging. This variability in plasma and brain vitamin C may then contribute to age-related cognitive differences.

As highlighted in previous studies, aging has been predominantly associated with significant reductions in plasma vitamin C levels. As with gender, both pharmacokinetic and/or lifestyle variability have been proposed to contribute to this aged-associated decline. Based on a range of animal and human studies that directly measured brain vitamin C, a reduction in vitamin C concentrations, as well as alterations in the vitamin’s absorption and distribution in the brain have been observed during aging. A series of proposed mechanisms may contribute to changes in brain vitamin C regulation and utilization during aging, these may include changes in brain hormones, oxidative stress, receptor distribution, astrocyte function, etc (Figure 3).

Collectively, this mini-review points to a possible effect of gender and age on the association between plasma/brain vitamin C and cognition. The reviewed literature suggests that there may be a link between gender differences in plasma/ brain vitamin C and gender-associated cognitive differences, particularly while females are pre-menopausal. However, it is still too early to conclude which cognitive abilities would be affected differently as a result of the gender vitamin C variation. Additionally, based on the reviewed literature, we can postulate that aged-associated differences in plasma/brain vitamin C may be linked with aged-associated cognitive differences, with older cohorts appearing more vulnerable to experience declines in plasma vitamin C concentrations alongside compromised distribution and regulation of the vitamin in the brain. Nonetheless, the results encourage future investigations to take into account both gender and age when assessing the link between plasma vitamin C and cognitive function. This will help increase our understanding of how gender and age-related differences in plasma and brain vitamin C may mediate differences in gender and age-related cognitive ability.

## Author Contributions

ASa and NT conceptualized the study in discussion with KR, AP, and IH. NT prepared the manuscript with contributions from all co-authors (KR, AP, ASa, IH, and ASc). All authors contributed to the article and approved the submitted version.

## Conflict of Interest

The authors declare that the research was conducted in the absence of any commercial or financial relationships that could be construed as a potential conflict of interest.
